# Safety and Feasibility of a Novel Exoskeleton for Locomotor Rehabilitation of Subjects With Spinal Cord Injury: A Prospective, Multi-Center, and Cross-Over Clinical Trial

**DOI:** 10.3389/fnbot.2022.848443

**Published:** 2022-05-12

**Authors:** Sijing Chen, Zhanbin Wang, Yongqiang Li, Jiashuai Tang, Xue Wang, Liping Huang, Zhuangwei Fang, Tao Xu, Jiang Xu, Feng Guo, Yizhao Wang, Jianjun Long, Xiaodong Wang, Fang Liu, Jianfeng Luo, Yulong Wang, Xiaolin Huang, Zishan Jia, Mei Shuai, Jianan Li

**Affiliations:** ^1^Center of Rehabilitation Medicine, The First Affiliated Hospital of Nanjing Medical University, Nanjing, China; ^2^Jiangsu Zhongshan Geriatric Rehabilitation Hospital, Nanjing, China; ^3^School of Automation Science and Electrical Engineering, Beihang University, Beijing, China; ^4^Department of Rehabilitation, The First Medical Center, Chinese PLA General Hospital, Beijing, China; ^5^Department of Rehabilitation Medicine, Tongji Hospital, Tongji Medical College, Huazhong University of Science and Technology, Wuhan, China; ^6^Department of Rehabilitation, Shenzhen Second People's Hospital, Shenzhen, China; ^7^Department of Rehabilitation, The First Affiliated Hospital of Shenzhen University, Shenzhen, China; ^8^Department of Biostatistics, School of Public Health, Fudan University, Shanghai, China; ^9^NHC Key Laboratory of Health Technology Assessment, Fudan University, Shanghai, China; ^10^Key Laboratory of Public Health Safety of Ministry of Education, Fudan University, Shanghai, China; ^11^School of Biological Science and Medical Engineering, Beijing Advanced Innovation Centre for Biomedical Engineering, Beihang University, Beijing, China

**Keywords:** exoskeleton, spinal cord injury (SCI), paraplegia, walking aid, rehabilitation, orthosis

## Abstract

**Objective:**

To evaluate the safety, walking efficiency, physiological cost, don and doff time cost, and user satisfaction of Ai-robot.

**Design:**

Prospective, multi-center, and cross-over trial.

**Subjects:**

Paraplegic subjects (*n* = 40) with T6–L2 level spinal cord injury.

**Methods:**

Subjects who could walk independently using Aiwalker, Ailegs, and hip knee ankle foot orthosis (HKAFO) for 6 min within 30 days of training underwent 10 sets of tests. In each set, they completed three 6-min walk test (6MWT) sessions using the three aids in random order.

**Results:**

Skin lesions, pressure sores, and fractures, were the main adverse events, likely due to a lack of experience in using exoskeleton systems. The average 6MWT distances of the Aiwalker, Ailegs, and HKAFO groups were 134.20 ± 18.74, 79.71 ± 18.06, and 48.31 ± 19.87 m, respectively. The average heart rate increases in the Aiwalker (4.21 ± 8.20%) and Ailegs (41.81 ± 23.47%) groups were both significantly lower than that in the HKAFO group (62.33 ± 28.32%) (both *p* < 0.001). The average donning/doffing time costs for Ailegs and Aiwalker were significantly shorter than that of HKAFO (both *p* < 0.001). Satisfaction was higher in the Ailegs and Aiwalker groups (both *p* < 0.001).

**Conclusion:**

Subjects with paraplegia below T6 level were able to ambulate safely and efficiently with Ai-robot. The use of Ai-robot should be learned under the guidance of experienced medical personnel.

## Introduction

Spinal cord injury (SCI) is a common cause of paralysis. The worldwide annual incidence of SCI varied from 13.0 to 220.0 per million people depending upon the country (Kang et al., 2017). Missing prevalence data for major populations persist and the range of reported global prevalence was between 440 and 526 per million (Fitzharris et al., 2013; Lee et al., 2013; New et al., 2015; Kang et al., 2017). Many people with SCI are confined to a wheelchair for life, causing a heavy burden to society and families. SCI can lead to limb paralysis and many complications such as osteoporosis, fractures, spinal deformities, muscle atrophy, cardiopulmonary dysfunction, obesity, and metabolic disorders, etc. (Castro et al., 1999; Giangregorio and McCartney, 2006; Shah et al., 2006; Liusuwan et al., 2007; Widman et al., 2007; Dudley-Javoroski, 2008; Adriaansen et al., 2012; Mulcahey et al., 2013; Lai et al., 2014; Sezer, 2015; Gee et al., 2019, 2021).

The advent of exoskeletal robotic technology can benefit the spinal cord injury population in three ways: (1) extensive repetitions of walking can help them improve and regain their walking ability, (2) the need for medical manual labor can be reduced, making extensive walking training feasible and even shortening the course of treatment, and (3) complications can be reduced, such as reduced pain, spasticity, osteoporosis and improved cardiorespiratory, lower urinary tract and bowel function (Esquenazi et al., 2012; Kolakowsky-Hayner, 2013; Benson et al., 2015; Stampacchia et al., 2016; Chun et al., 2019; Jang et al., 2019; Alashram et al., 2021; Brinkemper et al., 2021; Shackleton et al., 2021; Williams et al., 2021; Garnier-Villarreal et al., 2022).

**Figure 1:**
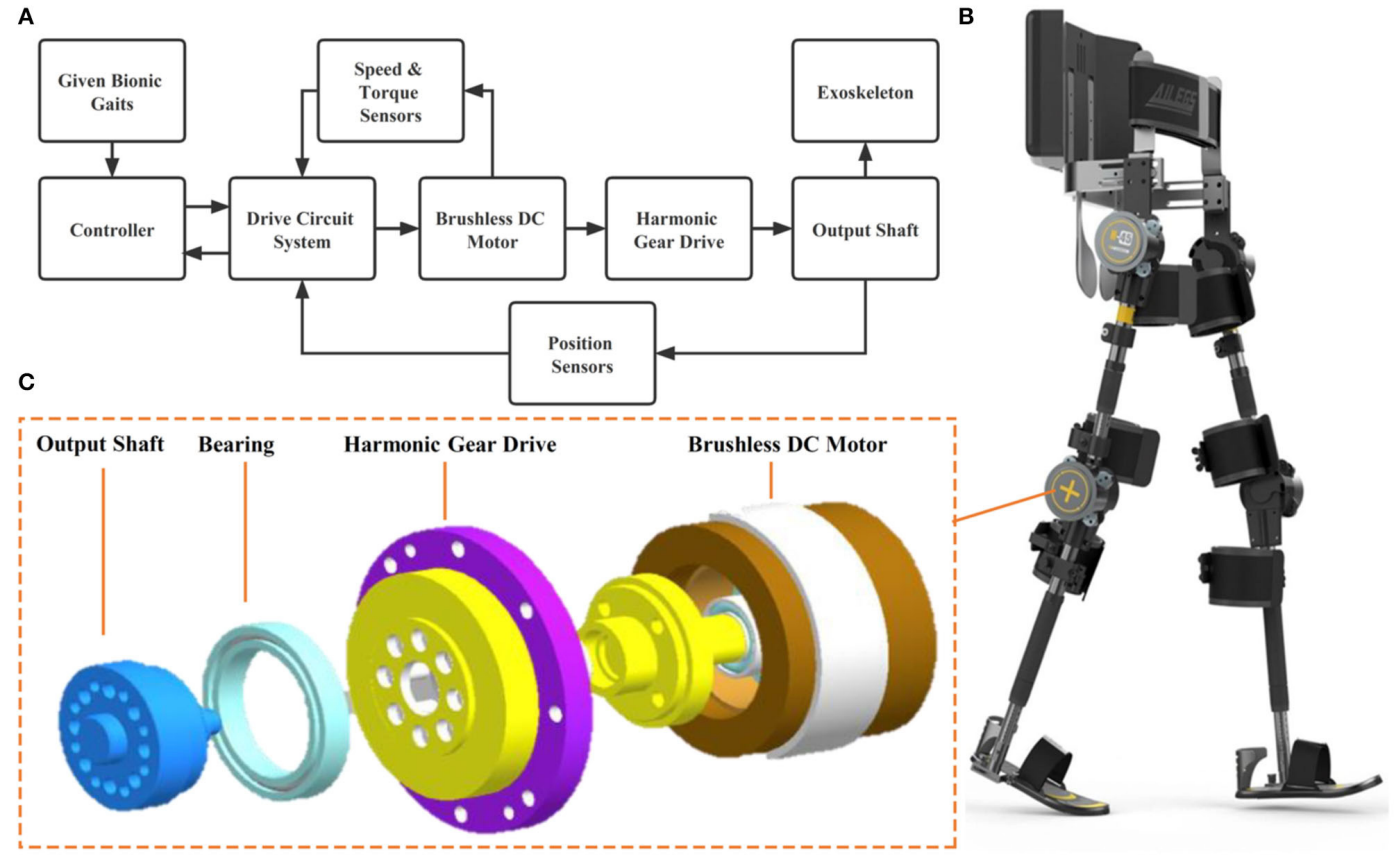
**(A)** Drive control system of Ai-robot, **(B)** Ailegs, and **(C)** motor structure.

**Figure 2:**
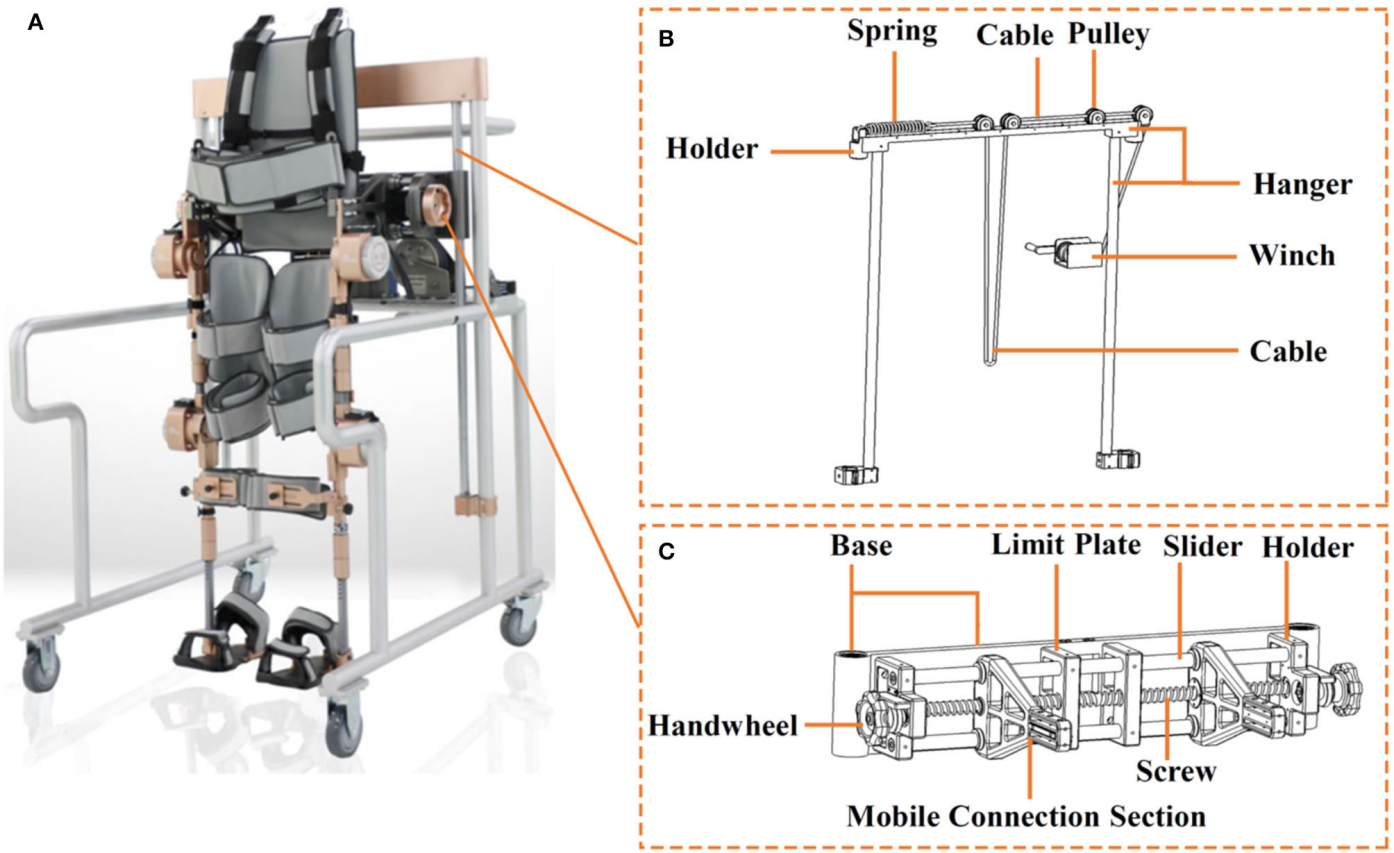
**(A)** Aiwalker, **(B)** suspension apparatus, and **(C)** adjustable waist support apparatus for Aiwalker.

Good fixation and support of the trunk during walking is required for subjects with poor upper limb and trunk function, insufficient endurance, or cognitive impairment. However, the existing stationary gait robots are often bulky and some are equipped with a treadmill (Peshkin et al., 2005; Bessler et al., 2020; Alashram et al., 2021; Calabrò et al., 2022). Patients mostly need to be transferred specifically to a dedicated treatment room to use the device, which cannot be used within the patient's ward, resulting in reduced accessibility. There are also devices that attach a robot exoskeleton to a mobile frame suspended in a sling with a high degree of freedom of trunk and pelvis movement. If the user cannot maintain balance with the trunk and lower extremities, the upper extremities are required to effectively hold the frame, which may not be suitable for patients with upper extremity paralysis and cognitive impairment (Bouri et al., 2006; Fukuda et al., 2015). Few exoskeleton robots are currently available to address these problems. For this reason, a new powered exoskeleton called Ai-robot has been developed (Figures 1B, 2A). It comes in two types, namely, Aiwalker and Ailegs.

Aiwalker is characterized by its small size and ease of mobility, allowing it to be moved directly to any ward or even bedside, and may also be suitable for use at home. The subject can transfer from the wheelchair to Aiwalker in a seated position and then adjust to a standing position after securing the straps. Walking exercises can be performed while suspended or on the real ground while speed and stride length could also be modified as needed. The subject sees the external environment as moving and changing, which enriches the input of visual signals, adds interest to the therapy and helps to improve mood. With the small footprint of the device, the Aiwalker may have a wide range of clinical applications, namely, patients with tetraplegia, cognitive impairment, ataxia, and those who cannot easily leave the ward. It may be suitable for home use for patients in the chronic phase, helping to reduce complications.

The Ailegs does not have a support platform and is suitable for patients with good upper limb and trunk function at the advanced stage of gait training. It requires elbow crutches to maintain balance when using it. The therapist can choose the appropriate type for the patient to provide a tailor-made treatment (Fukuda et al., 2015). Similar to other wearable exoskeletons, subjects can also walk outdoors, turn around, go through small obstacles such as speed bumps and go upstairs and downstairs with the assistance of Ailegs (Lajeunesse et al., 2015; Tefertiller et al., 2018).

The purpose of this first clinical research of Ai-robot was to evaluate the safety, walking efficiency, physiological cost, don and doff time cost, and user satisfaction.

## Materials and Methods

### Design

A prospective, multi-center, cross-over clinical trial was designed to assess the safety and effectiveness of two powered exoskeleton devices used to assist paraplegic subjects in walking by comparing them with a conventional hip knee ankle foot orthosis (HKAFO), based on subject walking ability indicators.

The clinical trial was conducted in four hospitals in eastern, northern, central, and southern China: The First Affiliated Hospital with Nanjing Medical University, Chinese PLA General Hospital, Affiliated Tongji Hospital with Tongji Medical College of Huazhong University of Science and Technology, and Shenzhen Second People's Hospital. The protocol, informed consent, case report form, and other implemented documents were approved by the ethical committee of the First Affiliated Hospital of Nanjing Medical University (No. 2017-MD-069). Registration was recorded at ClinicalTrials.gov (identifier: NCT 03452059).

Each participant was clearly informed of the purpose of the research and the potential benefits and risks of enrolling in the research. Informed consent was obtained from all individual participants included in the study.

Some subjects had previously walked with an HKAFO, but none had previously walked or stood with a powered exoskeleton walking aid. Subject eligibility criteria were as follows: (1) age 18–60 years, body weight <80 kg, height 1.55–1.85 m; (2) confirmed by MRI/CT, International Standards for Neurological Classification of Spinal Cord Injury (ISNCSCI): A–C (without walking ability), injury level T6–L2; (3) muscle tone (modified Ashworth Scale): ≤2; (4) passive range of motion (ROM) of the bilateral hip and knee joints approximately normal, while the bilateral ankle joint could be maintained in a neutral position; (5) muscle strength of upper limbs and physical strength sufficient to stabilize crutches during assisted walking; (6) muscle strength of upper limbs and physical strength sufficient to transfer independently between a wheelchair and Ailegs/Aiwalker device; (7) able to understand and actively participate in the training program, agreed and signed the informed consent form. Subjects with any of the following criteria were excluded (1) unable to walk due to severe joint ROM limitation; (2) unhealed spinal fractures and unstable clinical condition, consultation with orthopedists or other specialists if not fully confirmed; (3) skin injury or infection in the robot contact skin area or lower extremities; (4) subject showed poor compliance and was unable to complete the study in accordance with the requirements; (5) severe chronic obstructive pulmonary disease; (6) other contraindications or complications that may affect walking training; (7) severe cognitive or visual impairment; (8) unilateral neglect; (9) pregnant or lactating women; (10) unstable angina, severe arrhythmia, or other heart diseases.

### Devices

#### Common Design of Ai-Robot

Ai-robot (Ai-Robotics Technology Co. LTD, Beijing, China) uses a drive control system that provides closed-loop and coordinated motion control of the two hips and two knee motors (Figure 1C) simultaneously to achieve bionic gaits. The drive control system includes a controller, a driver (drive circuit system), a brushless DC motor, a harmonic gear drive, an information acquisition unit (relative coding disk and absolute coding disk), and an output shaft (Figure 1A). The controller is responsible for outputting the motion gait and coordinating the synchronized and coordinated motion among the four drives, while adjusting the motion parameters of the drives in real-time according to the state information such as the angular speed of the motor shaft and the angular position of the output shaft during the movement of the leg bars (Shuai, 2017). The rotation centers of the hip and knee motors should be on the same horizontal axis as the user's hip and knee joint rotation centers, respectively. The device is connected to the wearer's limb *via* straps on the waist, thigh, calf, and foot.

**Figure 3:**
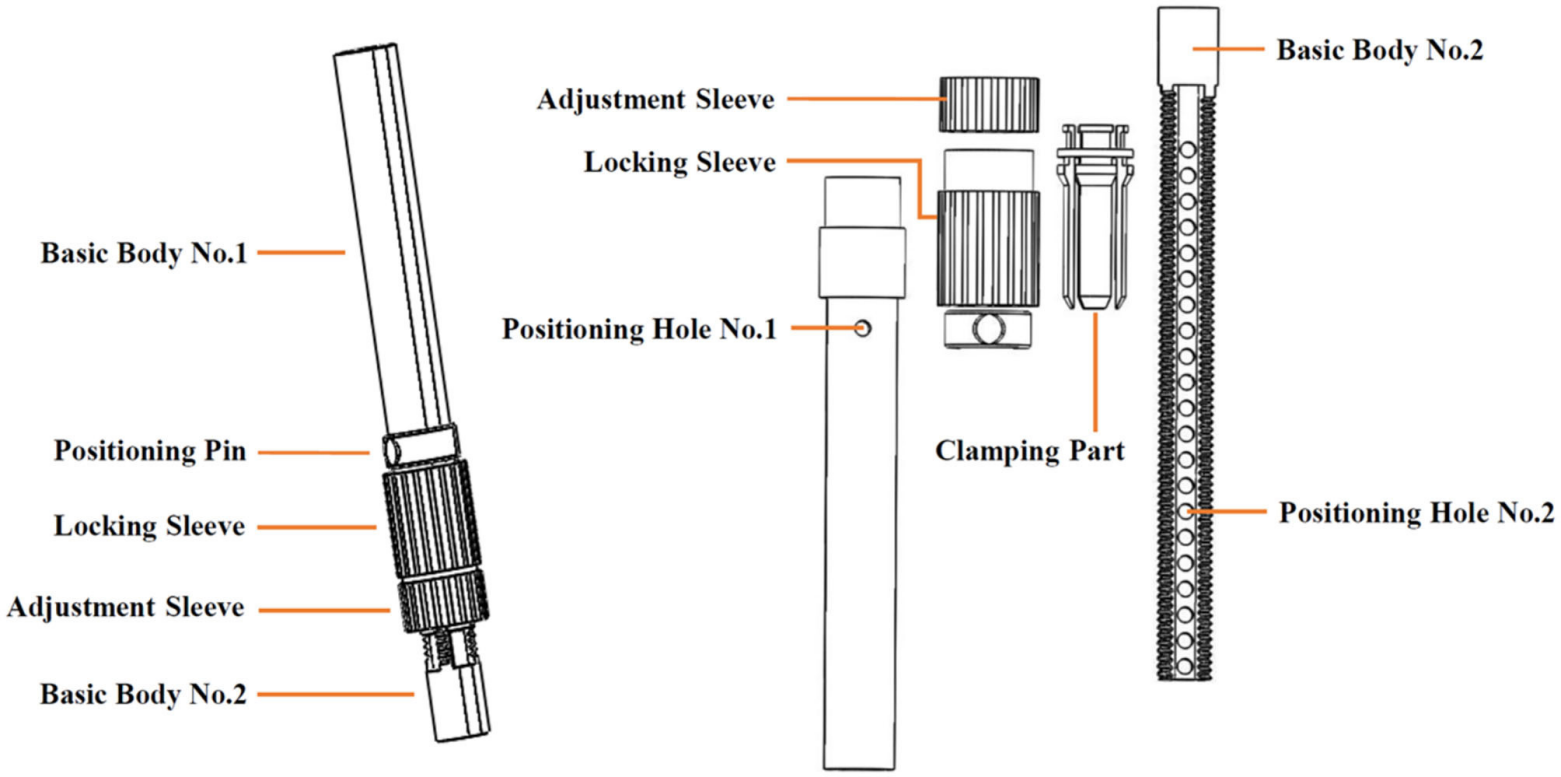
Retractable structure for Ai-robot leg bars.

Ai-robot provides sufficient power to assist walking without the need for active exertion of the lower limbs. The power is supplied by a 48 V 18,650 lithium battery with 15 Ah max capacity and 20 A max discharge current. The service life of Ai-robot is 8 years. The thigh and calf bars are designed as a retractable structure, with convenient and precise length adjustment for quick adaptation for users of different heights and body shapes (Figure 3) (Shuai, 2019).

The angles of the hip and knee joints can also be optimally adjusted to the needs of the user. The maximum angles are hip flexion 33°, hip extension 23° (Aiwalker)/0° (Ailegs), knee flexion 53°, knee extension 0°. The duration of each gait cycle can be adjusted between 2.45 and 5.25 s, as required. Its gait training control strategy is shown in Figure 1A, which takes the given bionic gait as the standard gait input to the drive control system, establishes the lower limb dynamics and kinematics model, calculates and controls the motion output, and drives the user's lower limb to perform the movement.

#### Type Design of Ai-Robot

**Figure 4:**
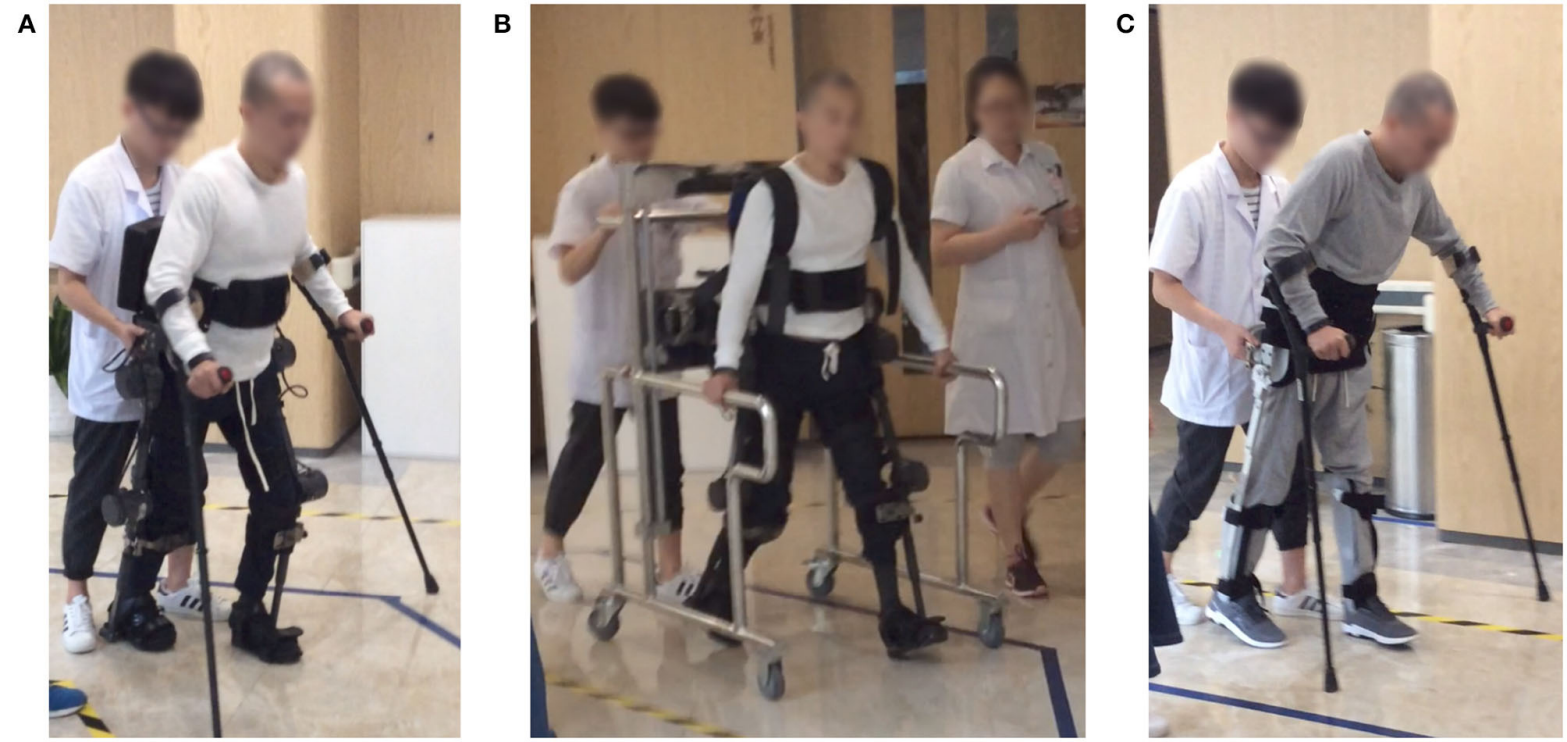
Trial devices. **(A)** Ailegs, **(B)** Aiwalker, and **(C)** HKAFO.

**Figure 5:**
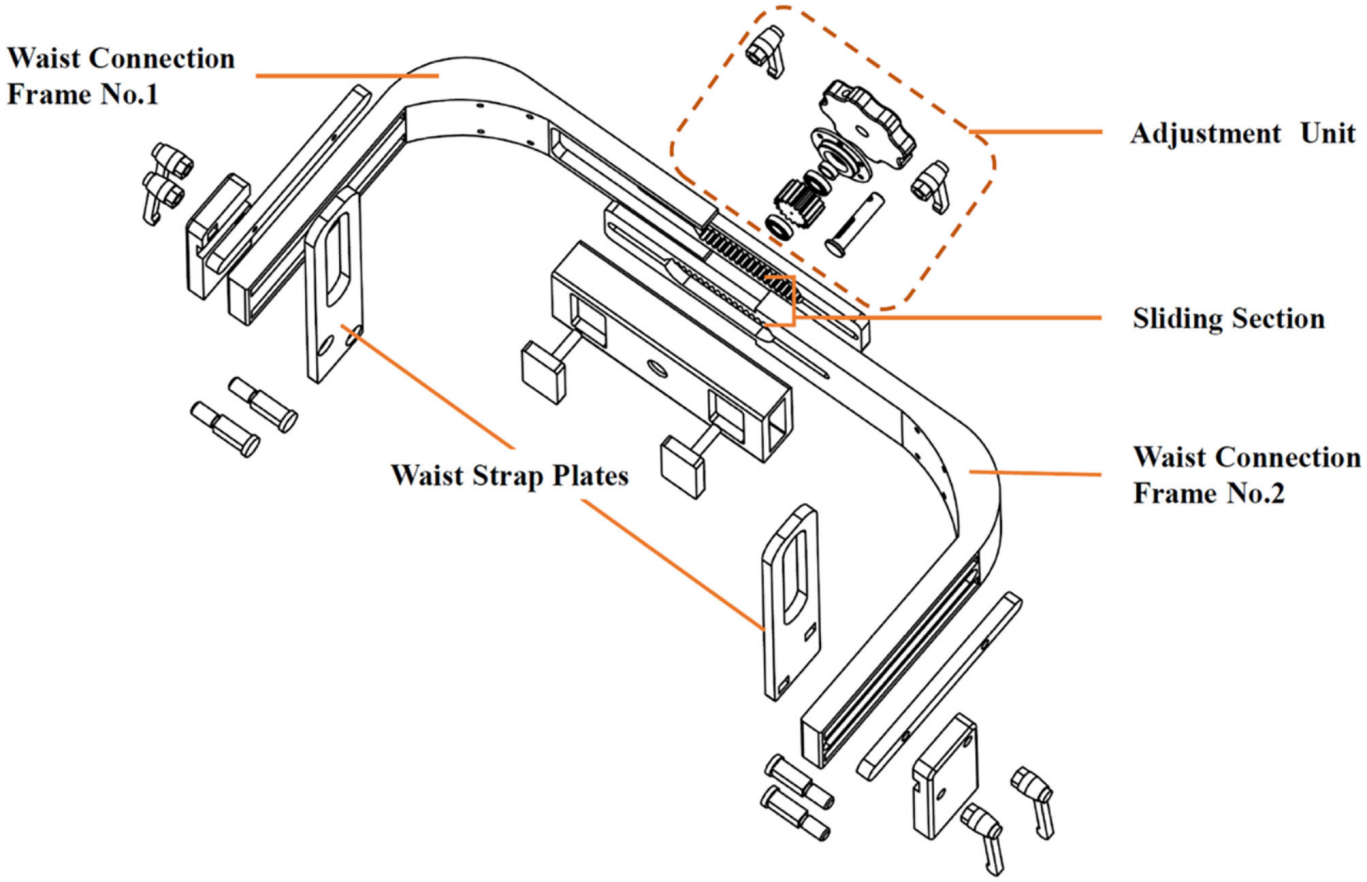
Adjustable waist apparatus for Ailegs.

Ailegs (frameless type of Ai-robot) weighs 25 kg and is made primarily of titanium alloy, requiring a pair of elbow crutches or walking aid for balance (Figure 4A). The waist structure connecting the lower limb exoskeleton comprises two waist connection frames and an adjustment unit. The waist structure connecting the lower limb exoskeleton comprises two first waist connection frames and an adjustment component unit. Each of the two waist connectors is provided with a sliding section, which is aligned with each other and connected to the adjustment unit. By turning the adjusting unit, the two connectors can be moved closer or further away from each other simultaneously, thus, adjusting the width of the waist structure (Figure 5) (Shuai, 2020).

Aiwalker (frame type of Ai-robot) weighs 80 kg and is mainly made of titanium alloy and stainless steel. It consists of a similar structure of Ailegs and a mobile support platform that provides stable trunk support. There are four wheels under the platform to allow easy movement of the device (Figure 4B). Its main features are waist fixation, lower limb drive, and true overground ambulation. The waist support device connected to the lower limb exoskeleton has an adjustable distance between the two mobile connection sections, thus being suitable for users with different waist widths (Figure 2C) (Shuai, 2018a). The suspension system shown in Figure 2B is capable of ensuring up and down movement of the human gravity center in the vertical plane during body weight support training or overground training. The suspension system includes a suspension frame, pulley, cable, holder, spring, winch, etc. The suspension structure will be locked when the lumbar support device is suspended to a predetermined position. The movement of the user's lower limbs also drives the waist support device up and down relative to the predetermined position (Shuai, 2018b). Aiwalker is designed to securely and stably fix the user's trunk and pelvis without requiring active effort to maintain standing balance.

#### HKAFO

Considering that SCI subjects with T6-L1 do not have the ability to flex the hips, the effect of walking with KAFO will be reduced. Therefore, HKAFO was chosen as a control. A custom-made unpowered HKAFO for each subject was produced by Beijing Sereborn Technology Co. LTD (Figure 4C).

### Experiment Protocol

This clinical trial was divided into the screening, trial, and data analysis phases. Since it was the first clinical use of Ai-robot, the therapist followed closely behind the subject during the Ailegs and HKAFO assisted ambulation to ensure safety. When using Aiwalker, the therapist walks behind the device and controls the direction of walking.

#### Screening Phase

During the screening phase, in addition to meeting the inclusion and exclusion criteria, subjects were assessed for their proficiency in independent transfer between wheelchair and walking aids, and ability to walk independently with Ailegs (with a pair of elbow crutches) and HKAFO (with a pair of elbow crutches). Only adaptive use was required for Aiwalker because it is a passive walking aid. If the subject was unable to independently walk with any of the three devices continuously for 6 min (due to lack of physical or trunk strength, postural hypotension, fear, etc.), a screening failure was recorded. The screening period did not exceed 30 days for each individual subject.

In total 49 participants signed informed consent forms, 40 were enrolled, and 9 failed the screening (2 were not exposed to the devices and 7 were unable to achieve the expected level of proficiency with the devices). As shown in Table 1, the mean age of the participants was 38.1 ± 9.4 years and injury levels ranged from T6 to L2.

**Table 1:** Demographic characteristics of participants.

**Characteristic**	**Value**
Sex (*n*)	Male (31), female (9)
Age (years), mean ± SD	38.1 ± 9.4
Height (cm), mean ± SD	169.8 ± 6.5
Weight (kg), mean ± SD	62.2 ± 7.8
Level of injury (*n*)	T6–L2: T10 (15), T12 (8), T9 (5), T8/T11/L1 (3 for each level), and T6/T7/L2 (1 for each level)
Type of injury (*n*)	Traumatic (33), Non-traumatic (7)
AIS classification (*n*)	A (29), B (4), C (7)
Skin integrity (*n*)	Intact (36), Broken but not affect use of the devices (4)
Spasticity in lower limbs (*n*)	Yes but not affect use of the devices (10), No (30)
Arrhythmia (*n*)	Normal or abnormal without clinical significance (40)
HRrest (beats/minute), mean ± SD	77.6 ± 8.3
Blood Pressure (mmHg), mean ± SD	Systolic (118.0 ± 11.7), Diastolic (74.7 ± 9.1)

*AIS, American Spinal Cord Injury Association Impairment Scale; HRrest, heart rate rest*.

#### Trial Phase

After the researchers judged that the subjects had mastered the trial devices, the subjects entered the test phase. The experimental period was 5 days, with one group of tests each half workday, and a total of 10 sets of tests. In each group, subjects were assigned to finish three 6-min walk tests (6MWTs) using Aiwalker, Ailegs, and HKAFO, respectively, in a random order. The circular walkway of a hospital hall (perimeter of ≥ 100 m) was set up as a test trail. Test trials were pre-marked on the ground. The 6MWT with each device was conducted at least 30 min apart, and the next test was performed after confirming that the subject's heart rate, blood pressure, and breathing had normalized. Six (15%) of the 40 subjects did not complete all 10 trial sets due to cystoscopy, adverse events, or requests to withdraw from the research.

### Data Collection

#### Safety Indicator

##### Adverse Events

The following adverse medical events were monitored and their relationship to the device used analyzed: incidence of falls, skin damage, joint injury, fracture, and other adverse events.

##### Blood Pressure

Blood pressure was measured twice using a calibrated medical electronic blood pressure monitor. The first upper limb blood pressure measurement was taken in a sitting position immediately before the beginning of the walking test. The second blood pressure was taken from the same upper limb in the same position 3 min after the end of the walking test. Subjects removed their walking aids and returned to the wheelchair after blood pressure measurement.

#### Validity Indicator

##### Primary Validity Indicator

###### Six Minutes Walking Distance.

The 6MWT was used to record the maximum walking distance within 6 min (Tappan et al., 2012). A circular walkway with a circumference ≥100 m was used as a training and test trail, and the walking path was marked on the ground in advance. Subjects were asked to walk as fast as they could at a comfortable and self-determined speed. There were ≥30 min between tests, and heart rate, blood pressure, and respiratory rate were also required to return to resting levels before the next test.

###### Average Percentage Heart Rate Increase.

A wireless single-channel medical electrocardiogram (ECG) recorder (Wearable ECG Recorder, Nanjing Xijian Information Technology Co., Ltd., China) was used to record the channel II ECG before and during the total 6MWT. %HRI was calculated as follows:


$$\begin{array}{l} \%\text{HRI}=\frac{\text{HRwalk }-\text{ HRbefore}}{\text{HRbefore}}\times 100 \end{array}$$

where “HRwalk” was the average heart rate determined by ECG between 120 and 330 s of the 6MWT (the steady phase) and “HRbefore” was the heart rate determined by a 1-min ECG recorded just before the start of a test, after sufficient rest, to calculate the average heart rate in the resting state.

##### Secondary Validity Indicator

###### The Borg rating of Perceived Exertion Scale.

Subjects were evaluated using the Borg RPE scale after each walking test (Heath, 1998). Each participant was assessed 10 times, by self-rating how tired they felt after walking with each of the three devices. RPE scores ranged from 6 to 20, with 6 indicating no effort and no fatigue at all, and 20 indicating maximum effort and exertion.

###### Time Cost of Donning and Doffing.

Two dedicated medical staff helped each subject to get into and take off the walking aids without helping transfer. The donning period began at the point the subject was ready to transfer from the wheelchair to the walking aid and ended when they were ready to stand up with the walking aid, including changing shoes for HKAFO. The doffing period was defined as the time from the point the subject was ready to take off the device until transfer back to the wheelchair, including placing the feet back on the pedals.

###### Satisfaction Questionnaire.

After each 6MWT, participants were asked to rate their satisfaction levels regarding device comfort, don and doff speeds, and stability during walking. Satisfaction was rated on the following five-point scale: (1) very satisfied, (2) satisfied, (3) fair, (4) dissatisfied, and (5) very dissatisfied. Data for very satisfied and satisfied were used to calculate the percentage of satisfaction.

### Statistical Methods

#### Statistical Design and Evaluation Methods

The hypotheses of this study were that (1) the 6MWT distance of both Ailegs and Aiwalker group was farther than the HKAFO group and (2) the average %HRI of both Ailegs and Aiwalker group were lower than the HKAFO group. Two primary validity indicators were set in this study. The hypotheses for both indicators need to be valid for the test group (Ailegs and Aiwalker) to be considered superior to the control group (HKAFO).

For the primary validity indicators, 10 consecutive groups of data were collected considering that each study subject was crossed over to the three orthoses. The mixed model considered grouping (Ailegs, Aiwalker, and HKAFO groups), order (ABC/ACB/BCA/BAC/CAB/CBA, 6 in total), time (5 days in the morning and afternoon, 10 time points in total), and the interaction of grouping and time. If the interaction test *p* > 0.1, the interaction term was removed and the model was refitted.

The safety analysis will be performed separately for the three groups (Ailegs, Aiwalker, and HKAFO).

#### Statistical Analysis

The number of subjects, average, standard deviation, minimum, and maximum were calculated for continuous variables. A number of examples and percentages were used to summarize categorical variables. For continuous variables, differences among the Ailegs, Aiwalker, and HKAFO groups were compared using repeated measured analysis of variance, paired *t*-test, or paired sign rank-sum test, according to the data distribution. For categorical variables, differences among the Ailegs, Aiwalker, and HKAFO groups were compared by paired chi-square test. Data were used from the participants who had completed at least one set of walking tests. Differences among the Ailegs, Aiwalker, and HKAFO groups were compared using a mixed model, and the confidence intervals of differences were calculated. Adjusted *p*-values of multiple comparisons were performed using the Benjamini–Hochberg procedure to control the false discovery rate (FDR) at 0.05. All the statistical analyses were performed using SAS9.3 (SAS Institute, Cary, NC, USA), and *p* < 0.05 was considered statistically significant.

## Results

### Safety Indicator

#### Adverse Events

There were no falls, increased pain, increased muscle tone, fatigue, or cardiovascular episodes. A total of four adverse events determined to be device-related occurred in four different subjects, including two cases of mild skin abrasion (lumbosacral region and left lumbar region), one case of bilateral heel pressure sores, and one case of calcaneal sinus compression fracture.

#### Blood Pressure

**Figure 6:**
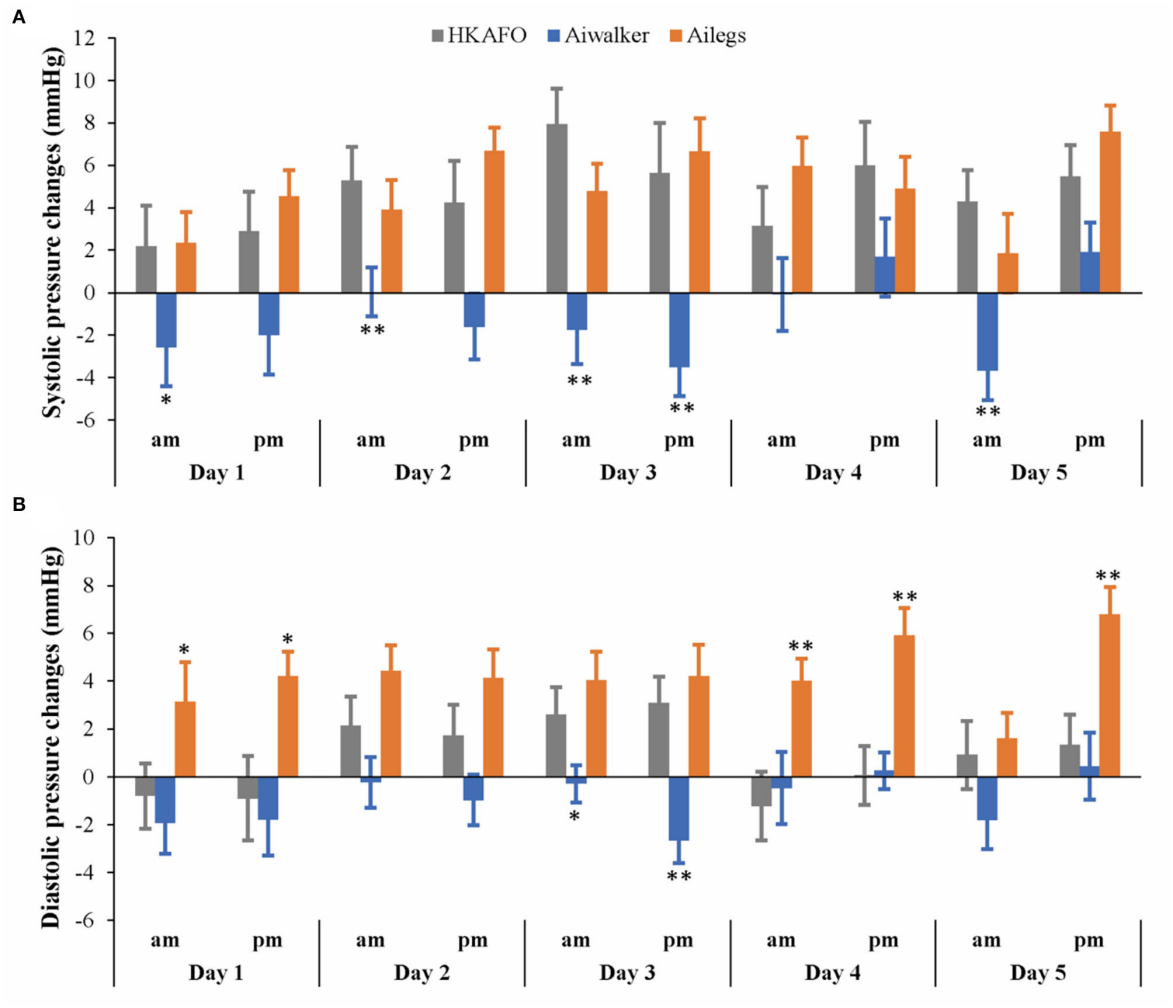
Systolic and diastolic pressure changes. **(A)** Systolic pressure changes, **(B)** diastolic pressure changes. *adjusted *p* < 0.05; **adjusted *p* < 0.01.

As shown in Figure 6, systolic blood pressure increased to varying degrees in the Ailegs (mean [SD] 4.89 [8.68] mmHg) and HKAFO (mean [SD] 4.67 [11.41] mmHg) groups after exercise; there was no significant difference between the two groups (Ailegs vs. HKAFO, the difference in LS means: −0.22 [95%CI, −1.58 to 1.14], adjusted *p* > 0.05, *n* = 40). In the Aiwalker group (mean [SD] −1.20 [9.85] mmHg), systolic blood pressure decreased slightly after exercise in 5 out of 10 tests; the difference with the HKAFO group was significant (Aiwalker vs. HKAFO, the difference in LS means: 5.87 [95%CI, 4.52–7.23], adjusted *p* < 0.001, *n* = 40).

After exercise, diastolic blood pressure in the Ailegs group (mean [SD] 4.22 [7.39] mmHg) was slightly higher than that in the HKAFO group (mean [SD] 0.89 [8.29] mmHg), and the difference was significant (Ailegs vs. HKAFO, the difference in LS means: −3.33 [95%CI, −4.39 to −2.27], adjusted *p* < 0.001, *n* = 40). Diastolic blood pressure decreased in the Aiwalker group (mean [SD] −0.97 [7.25] mmHg) and increased in the HKAFO group during most of the tests, and there was a significant difference between the two groups (Aiwalker vs. HKAFO difference in LS means: 1.86 [95%CI, 0.80–2.92], adjusted *p* < 0.001, *n* = 40).

### Validity Indicator

#### Primary Validity Indicator

##### Six Minutes Walking Distance

**Figure 7:**
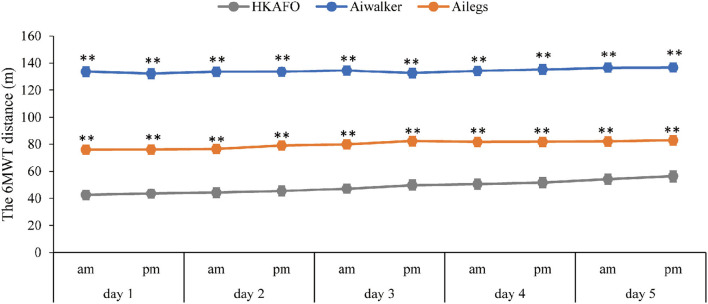
Six-minute walk test distance. **adjusted *p* < 0.01.

As shown in Figure 7, the mean (SD) 6MWT distance (m) of subjects in the Ailegs group was significantly farther than those in the HKAFO group (Ailegs 79.71[18.06] vs. HKAFO 48.31[19.87] m difference in LS means: 30.56 [95%CI, 28.42 to 32.70], adjusted *p* < 0.001, *n* = 40). Similarly, subjects in the Aiwalker group achieved greater 6MWT distances than those in the HKAFO group (Aiwalker 134.20[18.74] vs. HKAFO 48.31[19.87] m difference in LS means: 85.26 [95%CI, 82.94–87.59], adjusted *p* < 0.001, *n* = 40). The mean (SD) distance for the first 6MWT with HKAFO was 42.59 (19.22) m, and that of the tenth 6MWT was 56.33 (21.49) m, showing a trend of increasing speed. The mean distance of the first 6MWT using Ailegs was 76.04 (17.68) m, and the 10th was 82.84 (17.98) m, also showing an improving trend. As the subjects were walking fully passively, the 6MWT distance remained constant over the 10 tests in the Aiwalker group.

##### Average %HRI

**Figure 8:**
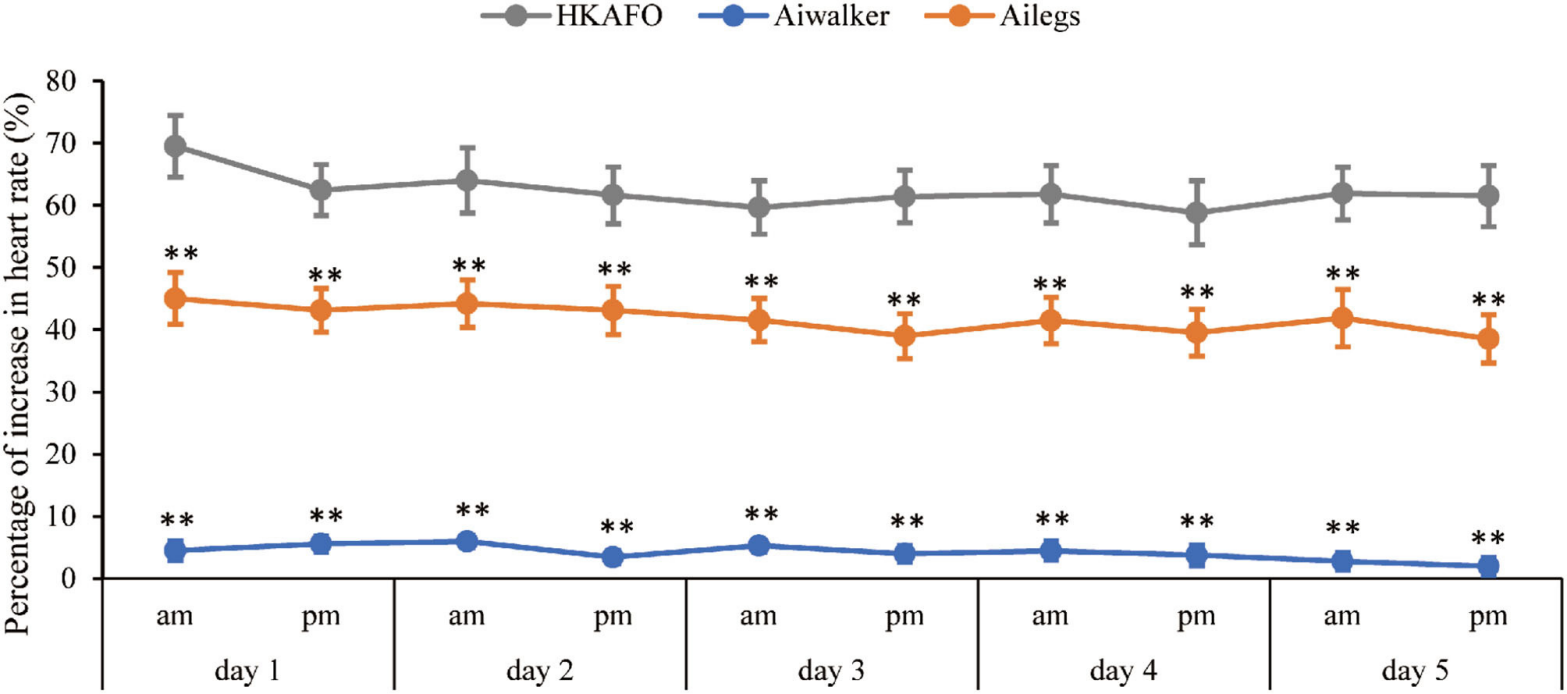
Percentage of increase in heart rate. **adjusted *p* < 0.01. %HRI = (HRwalk – HRbefore)/HRbefore × 100.

A slightly decreasing trend from test 1 to 10 of the %HRI in Ailegs and HKAFO groups was exhibited (Figure 8). The %HRI (mean ± SD) of 4.21 ± 8.20 in the Aiwalker group and 41.81 ± 23.47 in the Ailegs group were significantly lower than 62.33 ± 28.32 in the HKAFO group (Aiwalker vs. HKAFO, the difference in LS means: −58.7 [95%CI, −61.7 to −55.7], adjusted *p* < 0.001, *n* = 40; Ailegs vs. HKAFO, the difference in LS means: −22.2 [95%CI, −25.0 to −19.4], adjusted *p* < 0.001, *n* = 40).

#### Secondary Validity Indicator

##### Borg RPE Scale Scores

**Figure 9:**
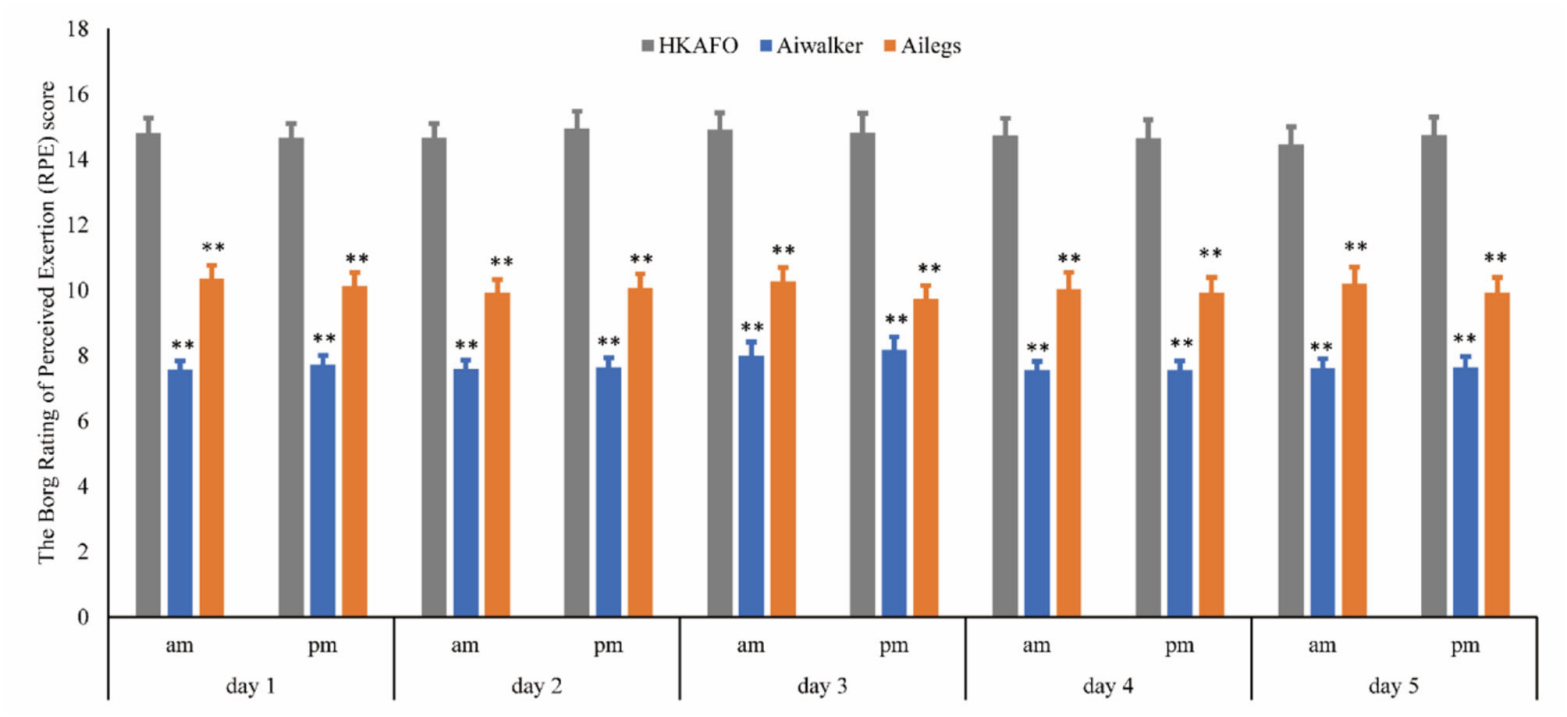
The Borg rating of perceived exertion score. **adjusted *p* < 0.01.

The RPE scores of subjects were relatively stable, with little variation. The mean (SD) scores were 10.07 (2.67), 7.71 (1.93), and 14.74 (3.17) in the Ailegs, Aiwalker, and HKAFO groups, respectively. The mean RPE scores for the Ailegs and Aiwalker groups were significantly lower than that of the HKAFO group (Ailegs vs. HKAFO, the difference in LS means: 4.68 [95%CI, 4.38–4.99], adjusted p < 0.001, *n* = 40; Aiwalker vs. HKAFO, the difference in LS means: 7.03 [95%CI, 6.73–7.33], adjusted *p* < 0.001, *n* = 40; Figure 9).

##### Time Cost for Donning and Doffing

**Figure 10:**
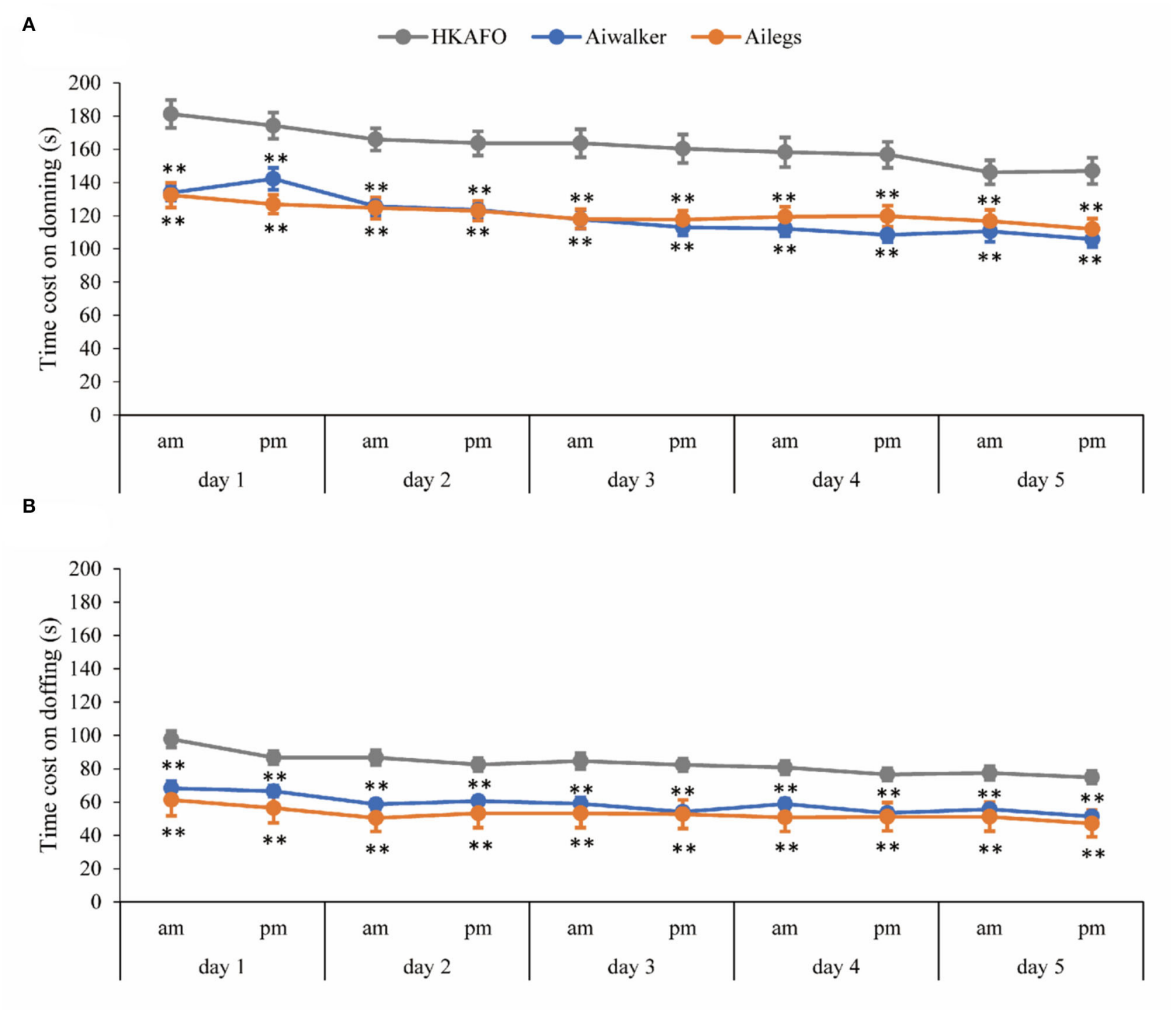
Time cost of donning and doffing. **(A)** Donning and **(B)** doffing. **adjusted *p* < 0.01.

The mean time costs for donning and doffing both Ailegs and Aiwalker were significantly shorter than that for HKAFO (*n* = 40, adjusted *p* < 0.001). In the 10 sets of tests, the time costs for the three devices all showed a shortening trend, and the doffing time cost was significantly shorter than that of donning. The time costs (mean ± SD) for donning were 121.24 ± 38.23 s (Ailegs), 119.82 ± 33.99 s (Aiwalker), and 162.23 ± 49.43 s (HKAFO). The mean time costs for doffing were 52.84 ± 21.77 s (Ailegs), 58.82 ± 20.66 s (Aiwalker), and 83.28 ± 26.70 s (HKAFO) (Figure 10).

##### Satisfaction

**Figure 11:**
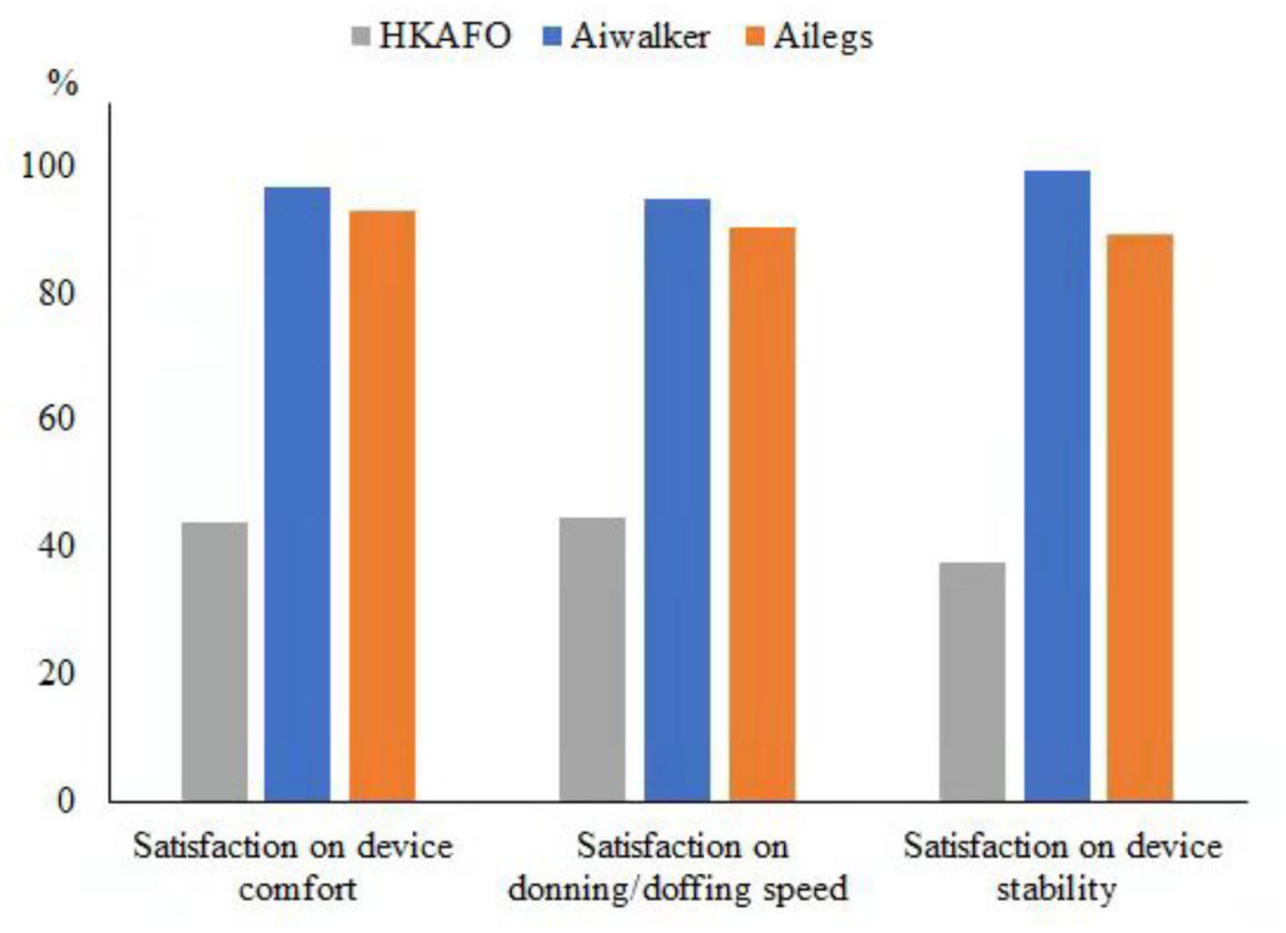
Satisfaction survey.

The results of the survey regarding device comfort, donning/doffing speed, and stability satisfaction all showed similar trends, with little change over the 10 sets of tests. The satisfaction of subjects was higher in both the Ailegs and Aiwalker groups than that in the HKAFO group (*n* = 40, all adjusted *p-*values of the comparisons were <0.001; Figure 11).

## Discussion

This study evaluated the safety, walking efficiency, and usability of a newly designed, easily mobile exoskeleton set that can be used in different stages of the disease. The results showed that the Ai-robot's technology is safe, but requires progressive weight-bearing on the lower extremities, with special attention to the skin at the heel and lumbosacral region. The Ai-robot assisted walking was more efficient, less physiological cost, faster in donning and doffing speed, and more satisfying for the user than traditional assistive walking devices.

We did not use ECG data from the entire 6MWT because subjects may be nervous at the beginning of the walk and the heart rate may be affected when they try to adapt to the equipment. Also, as the subjects approach the end of the walk, the heart rate may also be affected by emotion and the preparations of staff for measurements at the end. Therefore, we removed the ECG data from the first 2 min and the last 30 s and used the ECG data from the stable state in the middle segment to calculate heart rates.

A calcaneal sinus compression fracture occurred as a severe adverse event in this study. Due to loss of sensation in both lower extremities, the specific time point at which the injury occurred and the associated instrument could not be determined. After adaptive training using the exoskeleton robots and before training for HKAFO, the subject found that the skin temperature of their left calf was higher than that of the right calf in the late afternoon. Moreover, the left ankle was clearly bluish with swelling when he was cleaning his body in the evening. Compression fracture of the calcaneus sinus was confirmed by computed tomography. The likely reason may be a lack of experience with exoskeletons. The subject had not received lower limb weight bearing training for the last 4 years. SCI population has different degrees of osteoporosis below the injury level, particularly in the lower femur and the upper tibia. The fracture rate in the SCI population has been reported to be from 1% to 21% of subjects and may increase with time (Giangregorio and McCartney, 2006). The causes of osteoporosis may be related to factors such as nerve damage, loss of muscle loading, reduced mechanical stimulation, and duration of paralysis (Giangregorio and McCartney, 2006; Dudley-Javoroski, 2008; Johnston et al., 2008; Groah et al., 2010). Weight bearing of both legs in the upright position and weight bearing of one leg during walking will greatly increase skeletal stress. The calcaneus sinus has the lowest bone density in the calcaneus, and the occurrence of a compressible fracture could be explained by this mechanism. In this study, the researchers initially did not assess the weight-bearing capacity of the subject's lower extremities, nor did they gradually increase the amount and duration of weight bearing prior to adaptive training using the exoskeleton. The traditional dual-energy x-ray bone density test is performed on the proximal femur and lumbar spine and does not target the areas where spinal cord injuries are most likely to result in decreased bone density. Therefore, after this serious adverse event, two additional inclusion criteria were added: to confirm that the subject had the ability to bear weight in an upright position and to perform knee and ankle x-rays to confirm whether the bone density was severely reduced. Because of the inevitable decrease in bone density in the spinal cord injury population, the subject's ability to tolerate upright weight bearing is more important from our experience. Both lower limbs could tolerate the full weight-bearing position for at least 30 min before the use of the exoskeleton is suggested from this study.

In this study, there was also a case of pressure sore at the back of the heel. The reason was that the subject walked in shoes for the first time after SCI, and the shoes were new. He had a complete SCI with no lower limb sensory function. The shoe size was small and the material was stiff. While healthy people feel discomfort or pain in the heel when walking, people with SCI do not. The risk of pressure ulcers was reported in a clinical trial of Ekso, an exoskeleton-assisted walking device. The most common sites of skin erythema caused by pressure were the anterior tibia, greater trochanter of the femur, sacral region, abdomen, and dorsum of the feet (Kolakowsky-Hayner, 2013). These findings should serve as a reminder that skin color must be checked very carefully after each use of an exoskeleton device, particularly during the initial stages of use, due to lack of pain sensation.

Similar to the results of other studies, the 6MWT distance after wearing exoskeletons in this study was significantly higher than that when using the traditional non-dynamic orthosis, HKAFO; however, the walking distances in this research were shorter than those recorded in other ones. A meta-analysis by Miller et al. reported a summarized mean 6MWT distance of subjects wearing powered exoskeleton of 98 m (95% CI, 80–117 m) (Miller et al., 2016). While Arazpour et al. showed that the mean 6MWT distance was 120 ± 12.98 m in the exoskeleton group and 90.20 ± 10.63 m in the HKAFO group (Arazpour et al., 2012). This difference may be related to the duration of learning to use the exoskeleton devices and HKAFO during the screening period. The training period in our study included 4–6 training hours per day but was relatively short (no longer than 1 week), while training periods in other studies ranged widely from 1 to 24 weeks. Most experiments involved 60–120 min of training per session, three times a week (Esquenazi et al., 2012; Zeilig et al., 2012; Fineberg et al., 2013; Kolakowsky-Hayner, 2013; Kressler et al., 2014; Benson et al., 2015; Hartigan et al., 2015; Kozlowski et al., 2015; Yang et al., 2015). If the training period was extended in this research, subject walking distances may have increased. However, the purpose of this study was to verify the safety and feasibility of an exoskeleton walking aid, not the subject's improvement in walking speed and function.

Similar to other studies, this study also used mean %HRI to evaluate the physiological cost during walking (Arazpour et al., 2012). The physiological cost of walking with exoskeleton devices was lower than that with HKAFO, due to their power assist function. Walking distance with the exoskeleton was longer than that with HKAFO, further indicating that walking with an exoskeleton device consumed less energy. In a randomized controlled research, the physiological cost index was reported ~50% lower with a powered exoskeleton relative to HKAFO (Arazpour et al., 2012). The RPE scores for the walking aids used in this study were also consistent with the results of other studies, with the exoskeleton group scoring 9–11 and the HKAFO group 14–15 (Arazpour et al., 2012; Kolakowsky-Hayner, 2013; Kressler et al., 2014).

Although subjects did not show obvious changes in systolic and diastolic blood pressure before and after walking with Aiwalker and Ailegs, they did tend to have a slight decrease in systolic and diastolic blood pressure after Aiwalker use. Most of the subjects also stated that Aiwalker gave them a very stable and safe feeling and that they could relax and enjoy walking, which may explain the slight decrease in blood pressure. Research indicated a slight increase in blood pressure after walking with Rewalk, a comparable exoskeleton device, relative to before walking. The mean pre-training blood pressure was 121/77 (SD = 1.43/7.4) mmHg, while the mean post-training blood pressure was 129/83 (SD = 4.09/7.4) mmHg. Faulkner et al. conducted a pilot study on walking training assisted by an Ekso exoskeleton robot for the SCI population (Faulkner et al., 2019). The results showed that arterial wave reflection could be improved by a mean reduction of 9% and the training led to favorable changes in mean arterial pressure and central diastolic blood pressure, with mean decreases of 5 and 7 mmHg, respectively. These data suggest that exoskeleton robot-assisted walking training may be beneficial to the vascular health of subjects with SCI; this warrants further study.

The exoskeleton robotics industry is developing rapidly worldwide. Both Ailegs and Aiwalker were able to facilitate subjects walking on the real ground with a human-like gait. However, currently, Ai-robot cannot be controlled by the user to start, stop, sit down, stand up, etc. Further research is needed on (1) user control of the exoskeleton device, (2) personalized assistance provided by Ai-robot, and (3) the frequency and duration of training with Ai-robot, including gait speed and angle settings for each joint.

## Conclusion

Subjects with paraplegia (below T6 level) were able to walk safely and efficiently using the powered exoskeleton devices, Ailegs and Aiwalker, for overground ambulation with lower physiological cost. Satisfaction with Ailegs and Aiwalker was better than that with the traditional walking aid, HKAFO. The use of Ai-robot should be learned under the guidance of experienced medical personnel. The soft tissue compression at the strapping area and heel area needs to be checked after using the device. Subjects with SCI who have not recently trained to stand or walk will need to be weight-adapted before considering the use of an exoskeleton robot.

## Data Availability Statement

The raw data supporting the conclusions of this article will be made available by the authors, without undue reservation.

## Ethics Statement

The studies involving human participants were reviewed and approved by the Ethical Committee of the First Affiliated Hospital of Nanjing Medical University (No. 2017-MD-069). The patients/participants provided their written informed consent to participate in this study. Written informed consent was obtained from the individual(s) for the publication of any potentially identifiable images or data included in this article.

## Author Contributions

SC and ZW: manuscript writing. SC, YL, JT, XuW, LH, ZF, TX, JX, FG, YiW, JLo, XiW, and FL: subject recruitment and data collection. JLu and SC: data analysis. ZW and MS: design of the exoskeleton device. JLi, ZJ, XH, and YuW: study design and study supervision. All authors have read and approved the submitted version.

## Funding

This study was supported by Beijing Ai-Robotics Technology Co., Ltd, Beijing, China.

## Conflict of Interest

ZW and MS were employed by Beijing Ai-Robotics Technology Co., Ltd. The remaining authors declare that the research was conducted in the absence of any commercial or financial relationships that could be construed as a potential conflict of interest.

## Publisher's Note

All claims expressed in this article are solely those of the authors and do not necessarily represent those of their affiliated organizations, or those of the publisher, the editors and the reviewers. Any product that may be evaluated in this article, or claim that may be made by its manufacturer, is not guaranteed or endorsed by the publisher.

## References

[B1] Adriaansen J. J. E. van Asbeck F. W. A. Lindeman E. van der Woude L. H. V. de Groot S. Post M. W. M. (2012). Secondary health conditions in persons with a spinal cord injury for at least 10 years: design of a comprehensive long-term cross-sectional study. Disabil. Rehabil. 35, 1104–1110. 10.3109/09638288.2012.712196 22991949

[B2] Alashram A. R. Annino G. Padua E. (2021). Robot-assisted gait training in inspaniduals with spinal cord injury: a systematic review for the clinical effectiveness of Lokomat. J. Clin. Neurosci. 91, 260–269. 10.1016/j.jocn.2021.07.019 34373038

[B3] Arazpour M. Bani M. A. Hutchins S. W. Jones R. K. (2012). The physiological cost index of walking with mechanical and powered gait orthosis in patients with spinal cord injury. Spinal Cord 51, 356–359. 10.1038/sc.2012.162 23247013

[B4] Benson I. Hart K. Tussler D. van Middendorp J. J. (2015). Lower-limb exoskeletons for inspaniduals with chronic spinal cord injury: findings from a feasibility study. Clin. Rehabil. 30, 73–84. 10.1177/0269215515575166 25761635

[B5] Bessler J. Prange-Lasonder G. B. Schulte R. V. Schaake L. Prinsen E. C. Buurke J. H. (2020). Occurrence and type of adverse events during the use of stationary gait robots-a systematic literature review. Front. Robot. AI 7:557606. 10.3389/frobt.2020.557606 33501319 PMC7805916

[B6] Bouri M. Stauffer Y. Schmitt C. Allemand Y. Gnemmi S. Clavel R.. (2006). “The WalkTrainer: a robotic system for walking rehabilitation,” in 2006 IEEE International Conference on Robotics and Biomimetics (Kunming: IEEE).

[B7] Brinkemper A. Aach M. Grasmücke D. Jettkant B. Rosteius T. Dudda M.. (2021). Improved physiological gait in acute and chronic SCI patients after training with wearable cyborg hybrid assistive limb. Front. Neurorobot. 15:723206. 10.3389/fnbot.2021.723206 34512302 PMC8426634

[B8] Calabrò R. S. Billeri L. Ciappina F. Balletta T. Porcari B. Cannav,ò A.. (2022). Toward improving functional recovery in spinal cord injury using robotics: a pilot study focusing on ankle rehabilitation. Expert Rev. Med. Devices 19, 1–13. 10.1080/17434440.2021.1894125 33616471

[B9] Castro M. J. Apple D. F. Staron R. S. Campos G. E. R. Dudley G. A. (1999). Influence of complete spinal cord injury on skeletal muscle within 6 mo of injury. J. Appl. Physiol. 86, 350–358. 10.1152/jappl.1999.86.1.350 9887150

[B10] Chun A. Asselin P. K. Knezevic S. Kornfeld S. Bauman W. A. Korsten M. A.. (2019). Changes in bowel function following exoskeletal-assisted walking in persons with spinal cord injury: an observational pilot study. Spinal Cord 58, 459–466. 10.1038/s41393-019-0392-z 31822808 PMC7145720

[B11] Dudley-Javoroski S. (2008). Muscle and bone plasticity after spinal cord injury: Review of adaptations to disuse and to electrical muscle stimulation. J. Rehabil. Res. Dev. 45, 283–296. 10.1682/JRRD.2007.02.0031 18566946 PMC2744487

[B12] Esquenazi A. Talaty M. Packel A. Saulino M. (2012). The ReWalk powered exoskeleton to restore ambulatory function to inspaniduals with thoracic-level motor-complete spinal cord injury. Am. J. Phys. Med. Rehabil. 91, 911–921. 10.1097/PHM.0b013e318269d9a3 23085703

[B13] Faulkner J. Martinelli L. Cook K. Stoner L. Ryan-Stewart H. Paine E.. (2019). Effects of robotic-assisted gait training on the central vascular health of inspaniduals with spinal cord injury: a pilot study. J. Spinal Cord Med. 44, 299–305. 10.1080/10790268.2019.1656849 31525137 PMC7952073

[B14] Fineberg D. B. Asselin P. Harel N. Y. Agranova-Breyter I. Kornfeld S. D. Bauman W. A.. (2013). Vertical ground reaction force-based analysis of powered exoskeleton-assisted walking in persons with motor-complete paraplegia. J. Spinal Cord Med. 36, 313–321. 10.1179/2045772313Y.0000000126 23820147 PMC3758528

[B15] Fitzharris M. Cripps R. A. Lee B. B. (2013). Estimating the global incidence of traumatic spinal cord injury. Spinal Cord 52, 117–122. 10.1038/sc.2013.135 24322214

[B16] Fukuda H. Morishita T. Ogata T. Saita K. Hyakutake K. Watanabe J.. (2015). Tailor-made rehabilitation approach using multiple types of hybrid assistive limb robots for acute stroke patients: a pilot study. Assist. Technol. 28, 53–56. 10.1080/10400435.2015.1080768 26478988

[B17] Garnier-Villarreal M. Pinto D. Mummidisetty C. K. Jayaraman A. Tefertiller C. Charlifue S.. (2022). Predicting duration of outpatient physical therapy episodes for inspaniduals with spinal cord injury based on locomotor training strategy. Arch. Phys. Med. Rehabil. 103, 665–675. 10.1016/j.apmr.2021.07.815 34648804

[B18] Gee C. M. Eves N. D. Sheel A. W. West C. R. (2021). How does cervical spinal cord injury impact the cardiopulmonary response to exercise? Respir. Physiol. Neurobiol. 293:103714. 10.1016/j.resp.2021.103714 34118435

[B19] Gee C. M. Williams A. M. Sheel A. W. Eves N. D. West C. R. (2019). Respiratory muscle training in athletes with cervical spinal cord injury: effects on cardiopulmonary function and exercise capacity. J. Physiol. 597, 3673–3685. 10.1113/JP277943 31115056

[B20] Giangregorio L. McCartney N. (2006). Bone loss and muscle atrophy in spinal cord injury: epidemiology, fracture prediction, and rehabilitation strategies. J. Spinal Cord Med. 29, 489–500. 10.1080/10790268.2006.11753898 17274487 PMC1949032

[B21] Groah S. L. Lichy A. M. Libin A. V. Ljungberg I. (2010). intensive electrical stimulation attenuates femoral bone loss in acute spinal cord injury. PM R 2, 1080–1087. 10.1016/j.pmrj.2010.08.003 21145519

[B22] Hartigan C. Kandilakis C. Dalley S. Clausen M. Wilson E. Morrison S.. (2015). Mobility outcomes following five training sessions with a powered exoskeleton. Top Spinal Cord Inj. Rehabil. 21, 93–99. 10.1310/sci2102-93 26364278 PMC4568090

[B23] Heath E. M. (1998). Borg's perceived exertion and pain scales. Med. Sci. Sport Exer. 30:1461. 10.1249/00005768-199809000-00018

[B24] Jang Y.-C. Park H.-K. Han J.-Y. Choi I. S. Song M.-K. (2019). Cardiopulmonary function after robotic exoskeleton-assisted over-ground walking training of a patient with an incomplete spinal cord injury. Medicine 98:e18286. 10.1097/MD.0000000000018286 31852105 PMC6922438

[B25] Johnston T. E. Smith B. T. Oladeji O. Betz R. R. Lauer R. T. (2008). Outcomes of a home cycling program using functional electrical stimulation or passive motion for children with spinal cord injury: a case series. J. Spinal Cord Med. 31, 215–221. 10.1080/10790268.2008.11760715 18581671 PMC2565482

[B26] Kang Y. Ding H. Zhou H. Wei Z. Liu L. Pan D.. (2017). Epidemiology of worldwide spinal cord injury: a literature review. J. Neurorestoratol. 6, 1–9. 10.2147/JN.S143236

[B27] Kolakowsky-Hayner S. A. (2013). Safety and feasibility of using the Ekso^TM^ bionic exoskeleton to aid ambulation after spinal cord injury. J. Spine. S4, 1–8. 10.4172/2165-7939.S4-00328629390

[B28] Kozlowski A. Bryce T. Dijkers M. (2015). Time and effort required by persons with spinal cord injury to learn to use a powered exoskeleton for assisted walking. Top Spinal Cord Inj. Rehabil. 21, 110–121. 10.1310/sci2102-110 26364280 PMC4568092

[B29] Kressler J. Thomas C. K. Field-Fote E. C. Sanchez J. Widerström-Noga E. Cilien D. C.. (2014). Understanding therapeutic benefits of overground bionic ambulation: exploratory case series in persons with chronic, complete spinal cord injury. Arch. Phys. Med. Rehabil. 95, 1878–1887.e4. 10.1016/j.apmr.2014.04.026 24845221

[B30] Lai Y.-J. Lin C.-L. Chang Y.-J. Lin M.-C. Lee S.-T. Sung F.-C.. (2014). Spinal cord injury increases the risk of Type 2 diabetes: a population-based cohort study. Spine J. 14, 1957–1964. 10.1016/j.spinee.2013.12.011 24361350

[B31] Lajeunesse V. Vincent C. Routhier F. Careau E. Michaud F. (2015). Exoskeletons' design and usefulness evidence according to a systematic review of lower limb exoskeletons used for functional mobility by people with spinal cord injury. Disabil. Rehabil. Assist. Technol. 11, 535–547. 10.3109/17483107.2015.1080766 26340538

[B32] Lee B. B. Cripps R. A. Fitzharris M. Wing P. C. (2013). The global map for traumatic spinal cord injury epidemiology: update 2011 global incidence rate. Spinal Cord 52, 110–116. 10.1038/sc.2012.158 23439068

[B33] Liusuwan R. A. Widman L. M. Abresch R. T. Styne D. M. McDonald C. M. (2007). Body composition and resting energy expenditure in patients aged 11 to 21 years with spinal cord dysfunction compared to controls: comparisons and relationships among the groups. J. Spinal Cord Med. 30, S105–S111. 10.1080/10790268.2007.11754613 17874695 PMC2031969

[B34] Miller L. Zimmermann A. Herbert W. (2016). Clinical effectiveness and safety of powered exoskeleton-assisted walking in patients with spinal cord injury: systematic review with meta-analysis. Med. Devices (Auckl) 9, 455–466. 10.2147/MDER.S103102 27042146 PMC4809334

[B35] Mulcahey M. J. Gaughan J. Betz R. Samdani A. Barakat N. Hunter L. (2013). Neuromuscular scoliosis in children with spinal cord injury. Top Spinal Cord Inj. Rehabil. 19, 96–103. 10.1310/sci1902-96 23671379 PMC3641911

[B36] New P. W. Baxter D. Farry A. Noonan V. K. (2015). Estimating the incidence and prevalence of traumatic spinal cord injury in Australia. Arch. Phys. Med. Rehabil. 96, 76–83. 10.1016/j.apmr.2014.08.013 25218255

[B37] Peshkin M. Brown D. A. Santos-Munne J. J. Makhlin A. Lewis E. Colgate J. E.. (2005). “KineAssist: a robotic overground gait and balance training device,” in 9th International Conference on Rehabilitation Robotics (Chicago, IL: IEEE). 10.1310/tsr1502-131 18430678

[B38] Sezer N. (2015). Chronic complications of spinal cord injury. World J. Orthop. 6:24. 10.5312/wjo.v6.i1.24 25621208 PMC4303787

[B39] Shackleton C. Evans R. West S. Derman W. Albertus Y. (2021). Robotic walking to mitigate bone mineral density decline and adverse body composition in inspaniduals with incomplete spinal cord injury. Am. J. Phys. Med. Rehabil. 10.1097/PHM.0000000000001937. [Epub ahead of print]. 34864766

[B40] Shah P. K. Stevens J. E. Gregory C. M. Pathare N. C. Jayaraman A. Bickel S. C.. (2006). Lower-extremity muscle cross-sectional area after incomplete spinal cord injury. Arch. Phys. Med. Rehabil. 87, 772–778. 10.1016/j.apmr.2006.02.028 16731211

[B41] Shuai M. (2017). Drive Control System and the Exoskeleton Robot That Uses It. CN Patent No 206, 764, 764 U. Beijing: China National Intellectual Property Administration.

[B42] Shuai M. (2018a). Adjustable Waist Support Apparatus and the Exoskeleton Robot That Uses It. CN Patent No 108, 143, 593 A. Beijing: China National Intellectual Property Administration.

[B43] Shuai M. (2018b). Suspension Apparatus and the Exoskeleton Robot That Uses It. CN Patent No 108, 143, 584 A. Beijing: China National Intellectual Property Administration.

[B44] Shuai M. (2019). Retractable Structure and The Exoskeleton Robot That Uses It. CN Patent No 109, 431, 752 A. Beijing: China National Intellectual Property Administration.

[B45] Shuai M. (2020). Adjustable Waist Apparatus and the Exoskeleton Robot That Uses It. CN Patent No 111, 434, 323 A. Beijing: China National Intellectual Property Administration.

[B46] Stampacchia G. Rustici A. Bigazzi S. Gerini A. Tombini T. Mazzoleni S. (2016). Walking with a powered robotic exoskeleton: subjective experience, spasticity and pain in spinal cord injured persons. NeuroRehabilitation 39, 277–283. 10.3233/NRE-161358 27372363

[B47] Tappan R. Raad J. Moore J. (2012). Measurement characteristics and clinical utility of the 6-minute walk test among inspaniduals with spinal cord injury. Arch. Phys. Med. Rehabil. 93, 1675–1676. 10.1016/j.apmr.2012.07.005

[B48] Tefertiller C. Hays K. Jones J. Jayaraman A. Hartigan C. Bushnik T.. (2018). Initial outcomes from a multicenter study utilizing the indego powered exoskeleton in spinal cord injury. Top Spinal Cord Inj. Rehabil. 24, 78–85. 10.1310/sci17-00014 29434463 PMC5791927

[B49] Widman L. M. Abresch R. T. Styne D. M. McDonald C. M. (2007). Aerobic fitness and upper extremity strength in patients aged 11 to 21 years with spinal cord dysfunction as compared to ideal weight and overweight controls. J. Spinal Cord Med. 30, S88–S96. 10.1080/10790268.2007.11754611 17874693 PMC2031980

[B50] Williams A. Deegan E. Walter M. Stothers L. Lam T. (2021). Exoskeleton gait training to improve lower urinary tract function in people with motor-complete spinal cord injury: a randomized pilot trial. J. Rehabil. Med. 53:jrm00222. 10.2340/16501977-2864 34383958 PMC8638733

[B51] Yang A. Asselin P. Knezevic S. Kornfeld S. Spungen A. (2015). Assessment of in-hospital walking velocity and level of assistance in a powered exoskeleton in persons with spinal cord injury. Top Spinal Cord Inj. Rehabil. 21, 100–109. 10.1310/sci2102-100 26364279 PMC4568091

[B52] Zeilig G. Weingarden H. Zwecker M. Dudkiewicz I. Bloch A. Esquenazi A. (2012). Safety and tolerance of the ReWalkTMexoskeleton suit for ambulation by people with complete spinal cord injury: a pilot study. J. Spinal Cord Med. 35, 96–101. 10.1179/2045772312Y.0000000003 22333043 PMC3304563

